# Navigating ethical challenges of conducting randomized clinical trials on COVID-19

**DOI:** 10.1186/s13010-022-00115-3

**Published:** 2022-01-28

**Authors:** Dan Kabonge Kaye

**Affiliations:** 1 College of Health Sciences, Department of Obstetrics and Gynecology, Makerere University, P.O. Box 7072, Kampala, Uganda; 2 Johns Hopkins Berman Institute of Bioethics, Deering Hall, 1809 Ashland Avenue, Baltimore, MD 21205 USA

**Keywords:** COVID-19, Resource allocation, Clinical trials in emergency care, Ethical Challenges, Informed consent, Navigating Ethical Challenges

## Abstract

**Background:**

The contemporary frameworks for clinical research require informed consent for research participation that includes disclosure of material information, comprehension of disclosed information and voluntary consent to research participation. There is thus an urgent need to test, and an ethical imperative, to test, modify or refine medications or healthcare plans that could reduce patient morbidity, lower healthcare costs or strengthen healthcare systems.

**Methods:**

Conceptual review.

**Discussion:**

Although some allocation principles seem better than others, no single moral principle allocates interventions justly, necessitating combining the moral principles into multiprinciple allocation systems. The urgency notwithstanding, navigating ethical challenges related to conducting corona virus disease (COVID-19) clinical trials is mandatory, in order to safeguard the safety and welfare of research participants, ensure autonomy of participants, reduce possibilities for exploitation and ensure opportunities for research participation. The ethical challenges to can be categorized as challenges in allocation of resources for research; challenges of clinical equipoise in relation to the research questions; challenges of understanding disclosed information in potential participants; and challenges in obtaining informed consent.

**Conclusion:**

To navigate these challenges, stakeholders need a delicate balance of moral principles during allocation of resources for research. Investigators need to apply information processing theories to aid decision-making about research participation or employ acceptable modifications to improve the informed consent process. Research and ethics committees should strengthen research review and oversight to ensure rigor, responsiveness and transparency.

## Background

### The social value of randomized clinical trials (RCTs) for COVID-19

The COVID-19 pandemic presents the moral urgency to promptly conduct rigorous research than can generate evidence about the safety and efficacy of interventions to treat or prevent COVID-19 [1]. While a lot of information is available on clinical manifestations of COVID-19), significant gaps exist. For instance, there are gaps in knowledge about the disease transmission and treatment. While COVID-19 primarily spreads from person to person through respiratory droplets typically released when an infected person coughs or sneezes, it is unclear whether significant transmission occurs through the inhalation of aerosols (virions suspended in air), more since the virus may be aerosolized during certain activities (such as singing) and procedures such as intubation. Secondly, it is not clear why some infected individuals remain asymptomatic. There are also gaps in in knowledge about clinical manifestation, risk and prognostic factors of COCOD-19. It is unclear whether the duration of viral shedding is associated with severity of patients’ disease, or what factors are associated with viral shedding.

Regarding clinical disease, patients may present with several symptoms after an incubation period of 4–5 days, but factors that determine the clinical presentation are not clear. Symptoms include fever, cough, sore throat, malaise, myalgias, anosmia, ageusia, gastrointestinal symptoms (such as anorexia, nausea and diarrhea) [2, 3]. Shortness of breath usually signifies severe disease. While risk factors for complications of COVID-19 include older age (more than 65 years), cardiovascular disease, chronic lung disease, hypertension, diabetes and obesity [4, 5], it is unclear whether other comorbidities (such as kidney disease, immunosuppression and cancer) confer increased risk of complications, the evidence of worse clinical outcomes notwithstanding. Lastly, while there are no proven therapies for COVID-19, immunomodulating therapies such as glucocorticoids, convalescent plasma, and anticytokine therapy promise some benefit.

## Main text

### Challenges in priority setting for the research agenda on CVID-19

There are profound gaps in current knowledge about COVID-19 and yet the disease has severe health, social, and economic consequences, making it an imperative to conduct scientifically rigorous clinical research evaluating potential prevention, mitigating therapies, and supportive treatment options [1, 6]. The urgency for quick advances in COVID-19 treatment notwithstanding, there is an ethical imperative to obtain informed consent for participation in such RCTs. In the field of COVID-19 clinical research, several factors present ethical challenges to the conduct of RCTs. The randomized clinical trial is the gold standard for biomedical research involving human participants. The contemporary ethical framework for conduct of RCTs trials requires an informed consent for research participation. Thus, the decision to participate in RCTs is made by a competent individual after sufficient disclosure of the necessary material information (related to the condition and proposed interventions, including alternatives to participation), adequate understanding of this information, after which a voluntary decision is made. This decision should be in line with the participant’s preferences and values, and should not be subject to undue influence or manipulation, undue inducement or coercion.

There is marked uncertainty about the impact of COVID-19 on patients, including the best therapies to alleviate symptoms or treat patients. This challenge is compounded by the fact that the evidence-base evolves daily, and new information on epidemiological transmission, symptomatology, risk factors and prognostic factors evolved quite rapidly, making it difficult to document clinical equipoise throughout the research. Besides, the COVID-19 pandemic caused severe demands and shortages in the healthcare systems, to the extent that it threatens to overwhelm healthcare infrastructure. Also, the rapid rate of change makes it difficult to assess both the potential impact of alternative therapies and evaluate the potential mechanisms of action, side effects and complications of promising therapies [6]. The imperative to address the pandemic both generates urgent need for research into efficacious forms of treatment for COVID-19, and creates challenges on criteria for inclusion in clinical trials individuals who may be unable to provide informed consent due to several factors, which include the seriousness or acuteness of the disease and ongoing treatment.

Resource allocation is a central part of the decision-making process in the health care system owing to finite resources. The efficient and fair allocation of scarce medical resources is a major challenge in healthcare, necessitating trade-off between efficiency (such as medical need, ability to benefit) and fairness (such as waiting lists, equal chances), thus requiring a between multiple core ethical values. Contemporary ethical guidelines on how to approach such allocation decisions are guided by several underlying and often competing moral principles, such as utilitarianism (maximizing total benefits), egalitarianism (treating all equally), prioritarianism, (favoring some individuals on some criteria), and instrumentalism or reciprocity (promoting and rewarding social usefulness). The competing moral principles create challenges not only in the allocation of resources for healthcare, but also in priority setting for the research agenda. The first concern is decision of how much resources should be prioritized for research in comparison to other aspects such as prevention, treatment and vaccination. The considerations for setting any research agenda may include primary recommendations for maximizing benefits to the most-at-risk populations, by shifting research resources to patients at risk of severe disease and mortality (such as the elderly and those with co-morbidities), to prioritize patients with better prognosis or to focus resources on life years saved (that is, prioritizing the life of a younger patient in comparison to that of an older patient with a similar prognosis) [7, 8].

Equal treatment, maximizing benefits for all, prioritizing the worst off and instrumental value are ethically acceptable criteria for framing guidelines for resource allocation [9]. However, translating such criteria into explicit guidelines for research priority setting is challenging. Maximizing lives saved versus longevity, or using any other criteria, requires making judgment decisions that affect access to research opportunities and eventually treatment resources. Such decisions are therefore are subject to potential for discrimination against certain individuals (the poor, younger, older, weaker or minority), which may be morally unacceptable [7–9]. Still, prioritizing the majority poses risk of discrimination of the minority. Such discrimination, though unavoidable, requires development of guidelines to reduce or make such discrimination ethically justifiable or acceptable, and to balance inevitable concerns for maximizing prognosis and wellbeing with concerns of equity and social justice. Optimal use of any available resources and use of a fair and transparent allocation process are of critical importance during the COVID-19 pandemic [9] as they build trust in the healthcare system. Criteria that may be considered for resource allocation for research include likelihood of benefit from the research, possibility of treatment benefit in improving quality of life for patients, the urgency of a patient’s condition, and the resources required for successful treatment. Not conducting research compounds the risks of providing inadequate, ineffective, or even harmful care to patients with COVID-19.

### The ethical challenges to getting informed consent for RCTs

The main challenges of obtaining informed consent relate to disclosure of research related information, comprehension of disclosed information and ensuring a voluntary decision to participate. The concerns related to disclosure of information relate to how what foreseeable risks and potential harms should be disclosed, including an estimate of their likelihood as well as what steps will be taken to prevent, minimize them or mitigate against them. While any research involving investigational therapies or devices poses inherent unknown risks, COVID-19 poses many such risks considering that little research has been conducted on many aspects of COVID-19 infection and there is so much unverified information (some of it from research) on COVID-19 from both the clinical and research landscape. Absence of prior research on animal models makes it difficult to assess the effectiveness and safety of experimental drugs, devices or procedures on research participants [6] the fact that such interventions may have been approved for other indications on non-COVID populations notwithstanding [10, 11].

The additional ethical challenge to informed consent is uncertainty over key information necessary for an informed consent and challenges in disclosing alternatives. For participants with co-existing with cardiovascular, hematological or immunological comorbidities who are prescribed some of these medications or devices, or on whom research-related procedures are performed, providing accurate information on their continued risks or benefits (to enable them make an informed decision on the balance of benefits and risks) is challenging. Furthermore, there is a challenge of how to communicate the potential risks to facilitate an informed choice in the context of so much unverified information [12]. The discussion of risks to healthcare users should address both the probabilistic aspects and the importance and nature of the risk or adverse events being described [12]. How information about risks is interpreted depends on the stated probability of such event happening and how this information is communicated [12]. Risk is the probability that a hazard will give rise to harm [13]. Besides, there is need to disclose alternatives, yet for COVID-19, there are either very few or alternative therapies, depending on the setting and context.

As in other types of emergency research, potential participants considering participation in COVID-19 RCTs may be considered vulnerable [14]. However, such participation is ethically justifiable if the research meets the requirements for ethical clinical research (has social value, has sound methodological design, has fair subject selection, has procedures that maximize participant benefits while minimizing patient harms, is approved by an ethics committee and ensures respect for communities, among other requirements]15]. What participants understand from the disclosed information depends on not only on clear and robust disclosure from researchers, but also on both how this information is framed and the contextual factors. The factors that make it challenging for clinicians and researchers to meet their obligations to disclose research-related information in the context of COVID-19 also hinder patient comprehension, as there are so many sources of information about COVID-19 to which participants may have access. Besides, COVID-19 may affect an individual’s cognitive status, depending on the severity of the disease or any ongoing medication. How individuals perceive information varies depending on *subjective probability* -how individuals interpret information about risk and the objective probability-the actual harm, its severity for that given individual, and the outcome utilities (the importance or value placed on the event and individuals’ knowledge and experience.

While ensuring individual autonomy is important prior to research participation, autonomous decision making may not be feasible in patients with severe illness. Patients with moderate and severe illness may experience diminished capacity secondary to severe illness or ongoing medication (such as sedation in case of mechanical ventilation). Besides, patients may suffer severe anxiety, further clouding decision-making. In such a context, how the information on risk is framed matters. While different words with similar meaning, (such as adversity, complications, and burden) may be used to communicate severity of risk or potential harm, they may be understood differently by different individuals [12]. That is, may not be understood by (potential or real) participants in the way investigators may want them to be perceived or understood. Besides, since informed consent documents fail to promote decision-making [15], patients frequently make their decisions on participation in trials based on preformed ideas about research participation, often informed by experiences or their trust of the healthcare system in general and the investigators in particular. Also, the way information is presented verbally to patients matters [16]. Ideally, investigators should take adequate time present concise information during screening and assessment of eligibility, before any procedures like randomization [17]. Oftentimes, however, they provide inadequate information or pay inadequate attention to the process of decision-making during this process, compromising informed consent [15]. Moreover, patients’ perceptions of the manner and content of information disclosure are influenced by more general attitudes towards medical care, research, and institutions [18]. In addition, other challenges in the healthcare system such as staff shortages, long waiting times for patients before they are seen, and scarcity of personal protective equipment for patients, compound the challenges of the context and further compromise the informed consent process. Putting any guidance into practice is rather more complex and the way risk is framed may be persuasive or manipulative.

### Navigating the challenges to informed consent

RCTs are intended to generate evidence through testing hypotheses that generate scientifically acceptable conclusions that are applicable to benefit a wider patient population. While research and clinical care imply a very different thought process and design, they often occur together in emergency care research, can occur together. However, in this context, the boundaries of research and care are hazy. In the ICU setting, in particular, their boundaries may be hazy, considering that many medications and procedures used in critically ill patients may not have been tested and proven for this or any other populations] [4, 5, 10]. Besides, the complexity of the clinical situation often justifies unusual approaches, or the extreme severity of a patient’s illness may lead to extraordinary therapeutic measures [4, 5, 10]. This presents unique challenges for informed consent for research. To mitigate consenting challenges, the following actions could be employed: utilizing recruitment strategies that consider patients’ vulnerable status (such as by using information processing theories to design consent forms); utilizing acceptable alternatives to individual informed consent (such as community consultations, proxy consent, differed consent, targeted consent, and advanced consent); strengthening research review and oversight; and using a honest and transparent approach in the recruitment process.

### Engaging stakeholders

RCTs in healthcare are the gold standard for generating research evidence. However, there may be poor understanding among clinicians, investigators and other stakeholders of RCT methods and concepts (that is, equipoise, randomization, allocation, eligibility criteria, informed consent). There may be questions as to whether RCTs are the best way to spend money, particularly given the current competing needs in the healthcare system, which may make less and less funds available for research. Stakeholders engagement may be key in such a context [19, 20]. Such engaged stakeholders may include health systems, industry, purchasers, payers, policy makers, researchers, practitioners, patients or caregivers who are impacted by study findings. Within a research project, stakeholders may participate from conception to dissemination and perform roles beyond those of research participants (as funders, consultants, collaborators, co-investigators) and research users [21, 22]. Stakeholder engagement can add insight into steps and processes for development of research questions, study implementation, data analysis and dissemination of study findings, thereby increasing the prospect of making the study findings rapidly actionable, practicable, trustworthy and respected by the public [22]. Through stakeholder engagement, researchers can identify some of the reasons for poor or slow recruitment to RCTs, or potential reasons for discontinuation from research, from the perspectives of the community, patients, clinician, the trial sites, ethics committee members and the funders.

### Utilizing information processing theories to aid decision-making about research participation

Communication information about the research in the consent form, especially the purpose, study procedures and what potential benefits and risks are, in such a way that they are understood is key to enabling potential participants make informed decisions [23]. This raises questions as to what level of detail of information should be disclosed to enable an individual make informed decisions, as well as when and how this information should be provided. Because it reduces misunderstanding of trial methods and procedures, reinforces the understanding the potential benefits of RCTs, and increases assurance that participants use the disclosed information to make informed decisions, effective communication is essential for both for clinicians and their patients at the time of clinical trial recruitment [23]. One way to enhance understanding is to employ information-processing theories in the crafting of information presented in the informed consent forms. This approach employs the Fuzzy Trace Theory (FTT), a decision-making model drawn from cognitive science [24, 25]. FTT posits that presenting specific information, such as risk and benefit information in consent forms, does not itself directly influence a person’s reasoning, but rather, additional information is needed to link information surrounding potential risk to decision-making behavior [26].

When presented with risk information, individuals form two representations of that information into their working/ short-term memory [26]. The *verbatim form*, which represents the exact words, numbers or pictures, is transferred to working memory (where it rapidly diminishes and thus has limited impact on decision-making) [26]. The *gist form* represents the qualitative, essential meaning derived from the information and which is used for decision making. In a review of several studies of FTT and health-related outcomes, gist information was more strongly associated with decision making when compared to verbatim information alone [27]. If FTT promotes processing of the gist meaning of consent form information, rather than verbatim recall, it can assist individuals to make better informed choices about risk of research participation, particularly in high-risk studies [28]. Thus, the FTT may be used to develop patient decision aids, thereby making it easier to communicate basic RCT information like study purpose, potential risks, benefits, alternatives and key study procedures. Decision aids are designed to evoke a specific, deliberative process of decision-making about explicit choices from clearly described possibilities [15]. Patient decision aids not only present the information relevant to the decision, but also prompt decision makers to reflect on the different decision options, determine which matters are most important to them, and establish what additional information they need, providing a tool that organizes the entire process of decision making, rather than simply providing information [15].

### Strengthening research review and oversight by Research and Ethics Committees (RECs)

Identifying ways in which research can be undertaken ethically during emergencies is critical to promoting the contribution that ethically-conducted research can make to improving current and future emergency preparedness and response [29]. The function of ethical review by RECs or Institutional Review Boards (IRBs) is to ensure protection of participants from potential harms, in addition to ensuring beneficence and justice [29]. Considering that research involves risk and discomfort, beneficence translates into a risk/benefit analysis [30]. Beneficence refers to maximizing possible benefits and minimizing possible harms [29, 30]. Justice demands, among other things, that groups whose members bear the burdens of research stand benefit from such research, and research is not conducted with participants who are vulnerable unless it is to benefit them directly or benefit similar populations [29, 30]. Respect for persons requires that that individuals should be treated as autonomous agents and persons with diminished autonomy are entitled to protection [29, 30]. The several functions of informed consent include promotion of individual autonomy and rational decisions during self-determination, protection of patients and research participants, and avoidance of fraud, deception and coercion [29]. As a matter of justice, research is necessary to adapt existing ways of caring for patients and to ensure that the services provided, among other things, are effective, safe, appropriate and sensitive to their needs [29].

RECs should adopt values of rigor, responsiveness and transparency [29]. Rigor refers to consistently upholding of ethical principles through the values of equal respect, helping to reduce potential harms, promoting the safety and wellbeing of participants, and fairness in making decisions [29]. Responsiveness refers to timeliness in reaching ethical review and oversight decisions, as well as openness to adoption or adaptation of innovative research designs, as well as promoting consultations among stakeholders (researchers, funders and clinical care teams and affected communities) [29]. RECs should also promote equitable and responsible data sharing [29]. Equitable sharing requires systems that give researchers the same opportunities to access and use data irrespective of whether they are from low-income or high-income countries. Responsible sharing includes ensuring that investigators who access data and samples, once shared, maintain honesty and integrity while putting the data to optimum effect to help reduce suffering and promote wellbeing of patients. Transparency refers to employing clear and updated standard operating procedures in the review and oversight, which may involve more frequent meetings, adaptation of relevant technology like teleconferencing, electronic submission of research-related documents and clear communications of review decisions [29].

Regarding rigor, one aspect of concern to RECs is the issue of research on vulnerable individuals with moderate or severe COVID-19, and who for various reasons may be unable to understand disclosed consent information. The normative question is whether researchers should enroll participants who may not fully understand the information in the consent forms for COVID-19 studies [31]. Incomplete understanding of research raises concern of inability to utilize the disclosed information to develop a balance of potential harms and benefits in line with individuals’ preferences and values [31]. Also, individuals who do not understand that they are participating in research may be exposed to risks or may experience harms without realizing them to be occurring [31]. However, incomplete understanding should not necessarily forbid the conduct of research, nor should incomplete understanding carry equal concern in all types of research [32].

To ensure responsiveness, RECs should evaluate protocols on a case-by-case basis, based partly on potential risks posed by lack of understanding, especially the effect of the risk of such misunderstanding (on the participant or the community) and what the risks and benefits of the study are overall [32, 33]. To ensure both rigor and responsiveness, RECs need to employ different ethical principles to ensure that they protect the research subject from potential harm on one hand and ensure that nothing happens to the research subject without her valid consent on the other. In their mandate, RECs also ensure that research has both social value and is conducted ethically, whereby the potential harm of enrolling an individual in a clinical trial (possibly against their wishes or values, in case research participants are not in position to provide prior informed consent,) is fairly balanced against the potential good (to the participants, the population of patients or to the community), even when the individual may not benefit from the research) [32, 33]. From the principle of justice, the RECs are obligated to ensure that certain populations access opportunities to research or research outputs, the attendant risks to research participation notwithstanding. With regard to competent participants, the role of the REC should be that participants give a genuinely valid consent to participation in the research in question. For participants who are unable to provide prior consent, the RECs are obligated to ensure that adequate and appropriate safeguards are in place to achieve what would be needed to protect the participants from harm, to safeguard the rights of participants and to promote their wellbeing. While competent participants are in the best available position to analyze what risks are reasonable for them in line with their interests and values. The REC’s obligation does not end at approval of the research, but continues through the duration of the research, with oversight to ensure ongoing and protection of research participants from harm. Where the REC thinks the research is unnecessarily risky, (which includes research where participants are unable to provide prior consent), the measures to protect participants should be explicit, appropriate and reasonable [32, 33]. RECs should use their discretionary power to forbid or permit certain research while ensuring that research subjects are not harmed.

The relevance of rigor during review and oversight by RECs cannot be underestimated. Only fair inclusion criteria for participant recruitment in well-designed, high-quality RCTs with large enough sample sizes can contribute ethically to the discovery of safe and efficacious medications, procedures and devices for addressing the COVID-19 pandemic. RECs should take keen interest in the study design (or modifications thereof) as well as all study procedures. The potential harms from research participation may extend beyond the risk of receiving a previously untested intervention [34]. Also, keen interest should be taken to all study procedures, as ordinary non-therapeutic procedures are riskier depending on the severity of the participant’s condition [34]. For instance, research protocols may dictate a specific treatment protocol that limits the treating physician’s ability to tailor even nonexperimental interventions to specific patient needs [35]. Rigor in review may ensure that research is socially valuable and scientifically sound, that small repetitive poorly justified or non-rigorous studies that duplicate efforts and drain limited resources (without producing meaningful conclusions on the safety and efficacy of the interventions being tested) are not approved [29]. Study designs and procedures that render RCTs unable to answer the scientific questions of the study compromise validity and thereby evidence generated, and are thus ethically problematic (as they expose participants to avoidable risks without the prospect of benefit from generalizable knowledge) [29].

Regarding responsiveness, RECs should promote strategies that prevent participant exposure to unnecessary risks, ensure avoidance of exclusion of groups of research participants without an adequate justification (thus depriving them of the potential benefits of research), guarantee appropriate use of valuable research resources, promote integrity in investigators and build trust in the research enterprise [29]. It is the responsibility of RECs to ensure the balance of justice and beneficence. RECs should require that community consultations and stakeholder engagement precede recruitment of participants, and that relevant opinions of the different stakeholders are taken into account by investigators, before they approve modifications in study design, study procedures or informed consent processes [35]. This approach ensures a balance of beneficence and justice as perceived by all stakeholders (funders, investigators, patient representatives, clinicians and the community affected by the research). A question may be which individuals truly represent the community. Individual members of a homogeneous community, with a shared a set of values and opinions may provide a meaningful representation of that community, and may have representative views and values [35]. Investigators have an obligation to adopt culturally appropriate and respectful consent processes that demonstrate equal respect for participants [29].

The role of transparency for RECs needs to be emphasized. Ethics oversight should require mechanisms for registration of all clinical trials in registries are accessible to the investigators and the research stakeholders. Oversight should also ensure that mechanisms and guidelines to supervise COVID-19 clinical trials are strengthened. Transparency requires that the necessary action against non-compliance (which may include termination, suspension of recruitment and modification of trial procedures that are no longer justified or lack a favorable risk-benefit profile) is enforced in a fair and transparent manner.

### Utilizing acceptable modifications to individual informed consent

While recognizing several functions of informed consent [29], many ethical and legal justifications for the requirement of informed consent are based primarily on respect for personal autonomy or self-determination [36, 37]. Attention is placed on signing the consent form, with less attention to both the *process* of consent particularly understanding of risks and benefits and alternatives to a particular test or treatment [38, 39]. However, ethically valid consent should *be “a process of shared decision making based upon mutual respect and participation to guarantee both patient’s self-determination and personal well-being’* [38]. Thus, RECs should ensure that high-risk studies entail careful assessments and demonstration of participant understanding, especially of the potential harms involved [32]. If misunderstanding poses significant potential harms, even for studies with high baseline risk, individuals who do not fully understand the critical components of that clinical trial should be excluded from participation [32]. Conversely, for low-risk studies, it may be ethically acceptable for some individuals to be enrolled or to continue with participation even when they don’t understand the study, (especially) if the research might produce important generalizable results (without undue risk to trial subjects) [32]. Considering that vulnerability may refer to threats to self-development, self-determination, inclusion and equality (that may exist independent of research), such an approach ensures that vulnerable patients are offered both opportunities to participate and protection commensurate with their vulnerability [29].

#### Utilizing shared decision-making approaches

The shared decision-making (SDM) approach represents the middle way between a paternalistic approach (*the physician knows best)* and an autonomy-based approach *(the patient knows best)* [35, 40]. SDM’s goals are *“to make decisions in a manner consistent with the patient’s wishes”* [35, 41] and *“to respect patients as individuals and to deliver care consistent with their values and preferences”* [40]. This approach recognizes and values both investigator beneficence and participant autonomy; and thereby respects, protects, and supports the participants’ autonomous choices. SDM combines both information exchange (in which investigators provide research-based information about options and their risks and benefits and patients) and emphasizes the value-based preferences and choices (among options) made by the participants or their surrogates [35]. Informed consent and SDM have the same goal-to enhance the patient’s control over his or her medical care. While SDM developed primarily in ethics and informed consent developed primarily in law, both operate to create the same context and serve the same goal [35]. SDM is most appropriate in situations of uncertainty characterized by presence of 2 or more clinically reasonable alternatives, without compelling reasons for someone to choose one over the other [35]. Informed consent does not require the presence of clinical choices and may be appropriate as long as decisions to be made carry or entail significant risk [35]. In RCTs in emergency care research, decisions entail both risk and uncertainty, indicating that both SDM and informed consent are appropriate [35]. A broader view of SDM presumes a less individualistic view of personal autonomy, recognizing both positive and negative social impacts on self-determination [42]. It recognizes that patient preferences are often unclear, unsettled, changing, variable, and markedly depend on influences from context and relationships [42]. Also, the broad view recognizes that investigators may employ their own values and preferences to enhance participants’ appreciation of their options, preferences and rationales to arrive at the individual best choices in the given circumstances [42]. The broad view is aligned with *relational autonomy*, which recognizes that autonomous choices are generally achieved or realized over time in the context of positive and negative social relations [43].

SDM has several conceptual, normative, and practical challenges [44]. Its vagueness may threaten individual autonomy; some versions of SDM favor a division of labor, with investigators providing research-based factual information and participants adding their personal value-based preferences [40, 42, 44]. A joint decision, as a form of assisted decision making, may be reached through collaborative, conversational deliberation without emphasis on the patient’s basic legal and ethical rights to know and to decide [45–47]. Besides, poor communication skills among investigators [46], fundamental power differential between doctors and patients in clinical care or research [47, 48], lack of decision aids, and limited time available are a hinderance to SDM.

#### Dynamic consent for emergency care research

Voluntariness presents special challenges in emergency research (such as in the COVID-19 pandemic), yet emergency care is a critical entry pathway into the healthcare system and can generate data on triage, resuscitation and stabilization care for reducing preventable premature deaths [49–51]. While a truly informed consent process may be infeasible, there are alternative approaches, procedures and values that, through balancing of the ethical principles, could mitigate the threats to voluntariness for RCTs in emergency care research. Dynamic consent and reconsenting eligible participants may be employed, and RECs have an obligation, during oversight, to ensure that the appropriate measures are followed by the investigators. Dynamic consent is an approach to informed consent that enables on-going engagement and communication between individuals and investigators who are custodians of their data- a process that promotes accountability and transparency. In re-reconsenting and dynamic consent, a research participant (or a representative) makes a decision about whether to re-affirm a previous choice to participate in research [52]. Appropriate re-consenting approaches require that you notify subjects if new risks are identified, significant study alterations are made, or the initial consent process was inadequate. Re-consent should involve disclosure of any new, potentially material findings that could have emerged since initial consent, and therefore has potential to alter the availability, appreciation or understanding of options (especially harms and benefits) that are critical for an informed choice. However, there is uncertainty in determination of over which developments are important enough to disclose [6].

#### Targeted consent in emergency care research

Where trials interventions are within the standard of care procedures (such as in pragmatic RCTs) investigators may supplement appropriate consent for research only to the extent that the research differs from standard care [53]. Targeted consent ensures that patients are informed in general terms about research, (as details may introduce substantial selection bias and disruption of clinical care), and are given information to consent for only those interventions or procedures that lie outside standard care [53]. Thus, investigators thus obtain ethically appropriate consent with minimal additions to consent for standard care.

#### Deferred (or retrospective) consent in emergency research

In deferred consent, enrollment and initiation of research interventions occur without the patient’s prior consent [54]. Studies of this nature aim either to test treatments or to obtain tissue or samples from research participants in situations where the condition renders the participants incapable of providing prospective consent and the study cannot be conducted in any other population [54]. In a deferred-consent procedure, at the time of the experimental intervention or specimen collection, the patient is incapable of providing informed consent and their legal representative is incapable of providing consent or is not available. *Deferred consent* involves randomization at the investigator’s discretion according to explicit criteria followed by the request for patient’s (*deferred* subject *consent*) or representative’s (*deferred* proxy *consent*) informed *consent* at the earliest opportunity [54], when the patient is capable of providing informed consent or the representative is available [54]. Deferral of consent removes the patient from critical aspects of decision-making at a critical stage [55], so requires clear guidelines on how long one may remain in the study without prior consent [54], after which the patient’s participation is discontinued if consent is not obtained. Other requirements, if there therapeutic interventions, are that the treatment(s) under investigation should be potentially beneficial for the participants; there should not be any objection in advance to research participation; the research could not be conducted without the option of deferred consent; the evaluation of potential risks of the intervention do not exceed the risks from the standard treatment of the research participant; and the deferred-consent procedure has received prior approval from an ethics committee [54].

#### The consent substituted model in emergency care research

In pragmatic trials in emergency care, where investigators may be unable to get prior informed consent from participants, the consent substituted model [56] could be employed. In this approach, the values which an informed consent seeks to protect may be protected to some extent, as long as certain conditions are fulfilled: (1) responsiveness (the experimental intervention addresses an urgent medical need of the patients), (2) there is a foreseeable comparable risk-benefit ratio (, that is, the risk-benefit ratio of the experimental intervention is favorable, and at least as favorable as that of its comparator or any other available alternatives, (3) there is no conflicting preferences (that is, there is no evidence or compelling reason to suggest the individual’s participation in the research conflicts with enrolled patients’ preferences, interests or values), (4) minimal net risks (the nonbeneficial procedures employed in the study overall cumulatively pose no greater than minimal risk), and (5) prompt consent (consent for ongoing and additional emergency research interventions is obtained as soon as possible or feasible). These conditions, together, constitute an ethical substitute for informed consent in emergency research.

#### Advance consent in emergency care research

A novel approach to this situation is to use advanced consent. Advanced consent for research occurs when a potential participant is identified as being eligible for a study in the future and gives consent contingent on meeting the inclusion criteria at a later date, which could occur when the participant is no longer able to provide consent [57]. Advanced consent may be specific to a particular trial or may be a reflection of values to guide researchers in general about the patient’s desire to participate in research. Historically, this process has mainly been used for research in progressive diseases, such as dementia [58]. Though advanced consent may appear challenging to apply to emergency conditions given their unpredictable nature, there is still an opportunity to obtain advanced consent for research from populations with risk factors for certain emergency conditions.

#### Exception from informed consent in emergency care research

With exception from informed consent [59], research may be permitted when the therapy may directly benefit the research participant and it is not possible to conduct the research with informed consent. The conditions that should be satisfied to allow exception from informed consent [59] include presence of a life threatening condition as the context for the research question; the research protocol stipulates the therapeutic window of opportunity for the intervention as part of the research question; research cannot be conducted without waiver; subjects have prospect of benefit from the intervention; and the intervention procedure may be needed before consent is given such as in resuscitation research. Other conditions are that available therapies are unproven or unsatisfactory, risks associated with the research are reasonable in the context of the medical condition and in comparison, to standard therapy; and an ethics committee has approved the waiver, the study procedures and the consent documents. In addition, there should be community consultations and engagement, public disclosures of the research, presence of a data safety monitoring committee, and a plan to seek consent from proxies or legally authorized representatives [59].

### Implications for emergency care research

Emergency care research for COVID-19 should be considered an important social good, which, for individual participants, can provide access to new treatments or procedures unavailable in routine emergency care. It is therefore important that COVID-19 patients should not unfairly be denied access to research participation, which is detrimental to the interests of both the patients as a group and individual patients. Since no single ethical principle is satisfactory to incorporate all morally relevant considerations, allocation of research resources requires that individual principles should be combined into multi-principle systems. There are doubts whether the way research information is disclosed ensures comprehension and use of this information to make a balance of benefits and risks and eventual informed decisions about participation [60]. Methods for improving comprehension of information in the consent form include simplification of the language of the consent form, using audiovisual aids, reading the informed consent form with participants, asking participants to repeat study information in their own words, using open-ended questions to assess comprehension of the main messages (such as purpose, key study procedures, potential risks, benefits and alternatives), and encouraging the and potential research participant to ask questions [61–64]. While, in addition, conveying numerical probabilities of risk, using bullets in the informed consent document and motivated reasoning may have an important role [61–65], all these approaches have limitations in improving the informed consent process. Information processing theories may improve understanding of a shorter-design informed consent form.

Closer oversight before and during research is essential to ensure that the opportunity to benefit or contribute to the advancement of knowledge for other patients does not compromise safety and self-determination of the participants. Ethical review and oversight processes should promote agency and ethical inclusion (into research participation in emergency care research) of individuals, groups and communities. RECs should ensure that protocols have a balance of beneficence, autonomy and justice, that is, the population bearing the risks and burdens of research participation stands to benefit from the research and there is a favorable risk/benefit ratio, while autonomy is promoted to the greatest extent possible. RECs may advise on community engagement approaches or appropriate modifications to study procedures to minimize potential harms and risks. If community engagements are used, investigators have opportunity to publicly disclose the proposed research (its purpose, benefits, risks, and study procedures including opt out-processes) to community members, thus ensuring the social value and responsiveness of research to the needs of the COVID-19 patients. Communication of opt-out procedures enable subjects who do not want to participate (or to want to discontinue participation) to communicate that desire, thereby preserving individual autonomy. While the suggested mitigation approaches to individual consent do not preserve the right of self-determination of the research participant, they do provide an ethical means to advance autonomy, beneficence and justice. While there are genuine perceptions that informed consent may not be achievable [66, 67], there are ethically acceptable ways of seeking informed consent that try as much as possible to achieve a balance of beneficence, justice and autonomy.

## Data Availability

Not applicable.

## Abbreviations

FTT: Fuzzy Trace Theory
ICU: Intensive care unit
IRB: Institutional Review Board
RCT: Randomized Clinical trial
REC: Research and Ethics Committee
SDM: Shared decision making
